# Surgical wound infection caused by a multi drug resistant *Streptococcus pneumoniae* Serotype 19A after a total coloproctectomy with ileostomy

**DOI:** 10.11604/pamj.2020.35.97.19128

**Published:** 2020-04-06

**Authors:** Nzoyikorera Néhémie, Khalid Zerouali, Idrissa Diawara, Khalid Katfy, Fakhreddine Maaloum, Sylvestre Kabura, Bahija Zaki, Chehab Farid, Houria Belabbes, Naima Elmdaghri

**Affiliations:** 1 Department of Microbiology, Faculty of Medicine and Pharmacy, Hassan II University of Casablanca, Casablanca, Morocco; 2 Bacteriology-Virology and Hospital Hygiene Laboratory, Ibn Rochd University Hospital Centre, Casablanca, Morocco; 3 Faculty of Sciences and Health Techniques, Mohammed VI University of Health Sciences Casablanca, Morocco; 4 General Surgery Department III, Ibn Rochd University Hospital Center, Casablanca, Morocco

**Keywords:** Streptococcus pneumoniae, *serotype 19A*, *surgical wound infection*

## Abstract

*Streptococcus pneumoniae (S. pneumoniae)* colonizes asymptomatically the human nasopharynx. This pathogen is responsible for sinusitis, otitis media, pneumonia, bacteremia and meningitis. We report the case of a 35-year-old female patient who developed a surgical wound infection by a multi drug resistant *S. pneumoniae* serotype 19A after a total coloprotectomy. This first found in Morocco shows the implication of multidrug resistant *S. pneumoniae* in surgical wound infections.

## Introduction

*S. pneumoniae* is a common bacterium which colonizes the human nasopharynx. It is known for causing non invasive infections such as sinusitis, otitis media and invasive infections such as meningitis, pneumonia and bacteremia [1]. However, the recovery of this bacterium in soft tissue infections and surgical site infections is an uncommon found [2].

## Patient and observation

A 35-year-old female patient who underwent an ovarian transposition in 2017 and received 25 radiotherapy sessions was admitted to the general surgery ward of Ibn Rochd University Hospital Centre of Casablanca (UHC) for a rectal syndrome in a general deterioration context. At the rectal examination, a budding ulcer mass at 4cm from the anal margin was observed. After radiological, biological and anatomopathological assessment, the diagnosis of rectal adenocarcinoma was retained. Seven days after the total coloprotectomy, the bandage stained with pus and serous exudate output from the protective ileostomy was observed. A swab sample from the surgical wound was done and sent to the microbiology laboratory of Ibn Rochd Hospital for analysis. After 24 hours of incubation under 5% of CO_2_, bacteria grew on chocolate agar media and thioglycollate broth. The Gram´s staining revealed Gram-positive, lanceolate and capsulated diplococci while the catalase test on the colonies was negative. The culture on Columbia colistin-nalidixic acid agar media with 5% sheep blood showed after 24 hours of incubation, a pure culture of alpha hemolytic and optochin sensible streptococcus. Optochin sensitivity and bile solubility revealed the presence of *S. pneumoniae*.

The Real Time PCR targeting the autolysin genus *lytA* was performed to confirm the strain on CFX96 Real-Time System (Bio-Rad, Hercules, USA). The amplification curve confirmed the pneumococcus as the cause of the wound infection. The capsular typing was done by the procedures previously described by CDC [3] and adapted by Diawara for Moroccan isolates [4]. The strain belonged to serotype 19A. Antibiotic susceptibility tests were performed on Mueller-Hinton agar additioned with 5% of sheep blood (BioMerieux, Lyon, France) and interpreted according to the European Committee on Antimicrobial Susceptibility Testing (EUCAST 2018) recommendations [5]. Oxacillin, erythromycin, chloramphenicol, clindamycin, vancomycin, co-trimoxazole, rifampicin, tetracycline and levofloxacin were tested by disc diffusion. The minimum inhibitory concentration (MIC) of penicillin G, ampicillin and ceftriaxone were determined by E-test method. The strain was intermediate to ceftriaxone (1mg/L) and resistant to oxacillin, penicillin G (4mg/L), ampicillin (4mg/L), erythromycin, clindamycin and co-trimoxazole. It was only susceptible to levofloxacin, chloramphenicol and vancomycin. After seven days of betadine application and changing of bandages twice a day, the bandages remained clean and the serous exudate disappeared.

## Discussion

*S. pneumoniae* is a well-known major cause of multiple infections from non-invasive to invasive infections. In several countries, pneumococcal conjugated vaccines (PCV) have been recommended in childhood immunization programs for more than a decade. In Morocco, the PCV was introduced in the National Immunization Program in 2010 firstly by PCV 13, then switched to PCV 10 in 2012. PCV 13 targets the serotypes 3,6A and 19A in addition to those targeted by PCV 10 (4, 6B, 9V, 14, 18C, 19F, 23F, 1, 5 and 7F). The wound infection caused by this microorganism was surprising as it normally colonizes the human nasopharynx. Some authors reported the isolation of *S. pneumoniae* after surgical interventions[6,7]. In the large study conducted by Garcia-Lechuz and his colleagues on 3,201 isolates of *S. pneumoniae*, 69 strains (2.2%) were from skin and soft tissue infections, of which 11 strains were from surgical wound infections [2].

In many cases, the source of contamination is not well identified and still unknown. The nasopharynx of the surgeon was demonstrated as the source of contamination after an outbreak of 4 nosocomial-acquired surgical site infections as reported by Guillet [7]. The wound could be also contaminated by the patient himself, or his/her caregivers with pneumococcus carriage as suggested by Savini [6]. In our case, no investigations have been conducted on and around the patient nor the surgeon. We suggest that the healthcare staff may have the pneumococcus in carriage and may have contaminated the wound by droplets through sneezing or coughing while changing the bandage of the surgical wound. In Morocco, invasive pneumococcal diseases as well as the nasopharyngeal pneumococcal carriage are well documented [8,9]. Some pneumococcal isolates from wound infections such as burn infections or corneal abscess have been recorded in the microbiology laboratory of Ibn Rochd hospital (data not published). Nevertheless, there are no pneumococcus data in surgical wound infections.

This case is the first reported in our country. The serotype of our strain was 19A. This serogroup 19 was previously reported in the study of Garcia where the serotypes mostly isolated were 3, 19, 11 and 23 [2]. The main underlying conditions such as cancer, HIV infection and alcoholism have been reported in skin and soft tissue infections due to *S. pneumoniae* [2,7]. However, our patient suffered from rectal adenocarcinoma and did not receive pneumococcal vaccination. With regard to the antibiotic susceptibility, the multiresistance of our strain is alarming as bacteremia could occur secondary to surgical site infections as demonstrated by Guillet and his colleagues [7]. The strain isolated from surgical wound infection after a cat scratch disease was resistant to ampicillin, erythromycin, clindamycin and tetracycline [6]. However, the multiresistance in *S. pneumoniae* serotype 19A has already been documented [10]. In Casablanca, serotype 19A is known to be associated with multidrug resistance especially with penicillin resistance [8]. The patient´s wound was treated with betadine. This antiseptic has been used for decades to heal surgical wounds more than antibiotics because of its properties including the minimal risk of developing antibiotic resistance as well as the broad spectrum and persistent antimicrobial activity [11].

## Conclusion

This first report case in Morocco shows the unusual implication of *S. pneumoniae* serotype 19A in surgical wound infections. The large epidemiological study of *S. pneumoniae* in an adult population is needed to evaluate the indirect (herd) effect after the introduction of pneumococcal conjugate vaccine in the national immunization program since 2010 in Morocco.

## Competing interests

The authors declare no competing interests.

## References

[1] Chang MS, Woo JH (2016). The prevention of pneumococcal infections. Clinical and Experimental Vaccine Research.

[2] Garcia-Lechuz JM, Cuevas O, Castellares C, Perez-Fernandez C, Cercenado E, Bouza E (2007). Streptococcus pneumoniae skin and soft tissue infections: characterization of causative strains and clinical illness. European Journal of Clinical Microbiology & Infectious Diseases.

[3] CDC Alternative Multilocus Sequencing Typing Primers for S. pneumoniae. Streptococcus Laboratory.

[4] Diawara I (2014). Caractérisation phénotypique et génotypique des souches de Streptococcus pneumoniae isolées dans les infections invasives à Casablanca (Maroc).

[5] (2019). Société Française de Microbiologie.Streptococcus pneumoniae. CASFM / EUCAST.

[6] Savini V, Cecinati V, Onofrillo D, Consilvio NP, Polilli E, Crescenzi C (2012). Surgical Wound Infection by Streptococcus pneumoniae After a Cat-Scratch Disease. The International Journal of Lower Extremity Wounds.

[7] Guillet M, Zahar JR, Timsit MO, Grandin L, Carbonnelle E, Join-Lambert O (2012). Horizontal transmission of Streptococcus pneumoniae in the surgical ward: a rare source of nosocomial wound infection. American Journal of Infection Control.

[8] Diawara I, Zerouali K, Katfy K, Zaki B, Belabbes H, Najib J (2015). Invasive pneumococcal disease among children younger than 5 years of age before and after introduction of pneumococcal conjugate vaccine in Casablanca, Morocco. International Journal of Infectious Diseases.

[9] Warda K, Oufdou K, Zahlane K, Bouskraoui M (2013). Antibiotic resistance and serotype distribution of nasopharyngeal isolates of Streptococcus pneumoniae from children in Marrakech region (Morocco). Journal of Infection and Public Health.

[10] Isturiz R, Sings HL, Hilton B, Arguedas A, Reinert RR, Jodar L (2017). Streptococcus pneumoniae serotype 19A: worldwide epidemiology. Expert Review of Vaccines.

[11] Bigliardi PL, Alsagoff SAL, El-Kafrawi HY, Pyon JK, Wa CTC, Villa MA (2017). Povidone iodine in wound healing: a review of current concepts and practices. International Journal of Surgery.

